# Overcoming chemotherapy resistance using pH-sensitive hollow MnO_2_ nanoshells that target the hypoxic tumor microenvironment of metastasized oral squamous cell carcinoma

**DOI:** 10.1186/s12951-021-00901-9

**Published:** 2021-05-26

**Authors:** Zhi-hang Zhou, Si-yuan Liang, Tong-chao Zhao, Xu-zhuo Chen, Xian-kun Cao, Ming Qi, Ying-ying Huang, Wu-tong Ju, Meng Yang, Dong-wang Zhu, Yi-chuan Pang, Lai-ping Zhong

**Affiliations:** 1 Department of Oral and Maxillofacial-Head and Neck Oncology, Ninth People’s Hospital, College of Stomatology, Shanghai Jiao Tong University School of Medicine, No. 639 Zhizaoju Road, Shanghai, 200011 China; 2 Shanghai Key Laboratory of Stomatology, Shanghai Research Institute of Stomatology, National Clinical Research Center of Stomatology, Shanghai, China; 3 Department of Oral Surgery, Ninth People’s Hospital, College of Stomatology, Shanghai Jiao Tong University School of Medicine, Shanghai, China; 4 Department of Orthopaedics Surgery, Ninth People’s Hospital, College of Stomatology, Shanghai Jiao Tong University School of Medicine, Shanghai, China; 5 Shanghai Key Laboratory of Orthopaedic Implants, Shanghai, China; 6 Key Laboratory of Nuclear Physics and Ion-Beam Application (MOE), Fudan University, Shanghai, China; 7 Department of Nuclear Medicine, Fudan University Shanghai Cancer Center, Shanghai, China; 8 Center for Biomedical Imaging, Fudan University, Shanghai, China; 9 Department of Clinical Immunology, Ninth People’s Hospital, Shanghai Jiao Tong University School of Medicine, Shanghai, 200011 People’s Republic of China; 10 Department of Nuclear Medicine, Tenth People’s Hospital, Tongji University School of Medicine, No.301 Yanchang Middle Road, Shanghai, 200072 China

**Keywords:** MnO_2_, Tumor microenvironment, Oral squamous cell carcinoma, Hypoxia, Angiogenesis, Chemotherapy

## Abstract

**Background:**

Smart nanoscale drug delivery systems that target acidic tumor microenvironments (TME) could offer controlled release of drugs and modulate the hypoxic TME to enhance cancer therapy. The majority of previously reported MnO_2_ nanostructures are nanoparticles, nanosheets, or nanocomposites incorporated with other types of nanoparticles, which may not offer the most effective method for drug loading or for the controlled release of therapeutic payloads. Previous studies have designed MnO_2_ nanoshells that achieve tumor-specific and enhanced combination therapy for localized advanced cancer. However, the therapeutic effect of MnO_2_ nanoshells on metastatic cancer is still uncertain.

**Result:**

Here, intelligent “theranostic” platforms were synthesized based on hollow mesoporous MnO_2_ (H-MnO_2_) nanoshells that were loaded with chemotherapy agents docetaxel and cisplatin (TP) to form H-MnO_2_-PEG/TP nanoshells, which were designed to alleviate tumor hypoxia, attenuate angiogenesis, trigger the dissolution of Mn^2+^, and synergize the efficacy of first-class anticancer chemotherapy. The obtained H-MnO_2_-PEG/TP nanoshells decomposed in the acidic TME, releasing the loaded drugs (TP) and simultaneously attenuated tumor hypoxia and hypoxia-inducible factor-1α (HIF-1α) expression by inducing endogenous tumor hydrogen peroxide (H_2_O_2_) decomposition. In vitro experiments showed that compared with the control group, the proliferation, colony formation and migration ability of CAL27 and SCC7 cells were significantly reduced in H-MnO_2_-PEG/TP group, while cell apoptosis was enhanced, and the expression of hypoxia-inducible factor-1α(HIF-1α) was down-regulated. In vivo experiments showed that tumor to normal organ uptake ratio (T/N ratio) of mice in H-MnO_2_-PEG/TP group was significantly higher than that in TP group alone (without the nanoparticle), and tumor growth was partially delayed. In the H-MnO_2_-PEG/TP treatment group, HE staining showed that most of the tumor cells were severely damaged, and TUNEL assay showed cell apoptosis was up-regulated. He staining of renal and liver sections showed no obvious fibrosis, necrosis or hypertrophy, indicating good biosafety. Fluorescence staining showed that HIF-1α expression was decreased, suggesting that the accumulation of MnO_2_ in the tumor caused the decomposition of H_2_O_2_ into O_2_ and alleviated the hypoxia of the tumor.

**Conclusion:**

In conclusion, a remarkable in vivo and in vitro synergistic therapeutic effect is achieved through the combination of TP chemotherapy, which simultaneously triggered a series of antiangiogenic and oxidative antitumor reactions.

**Graphic abstract:**

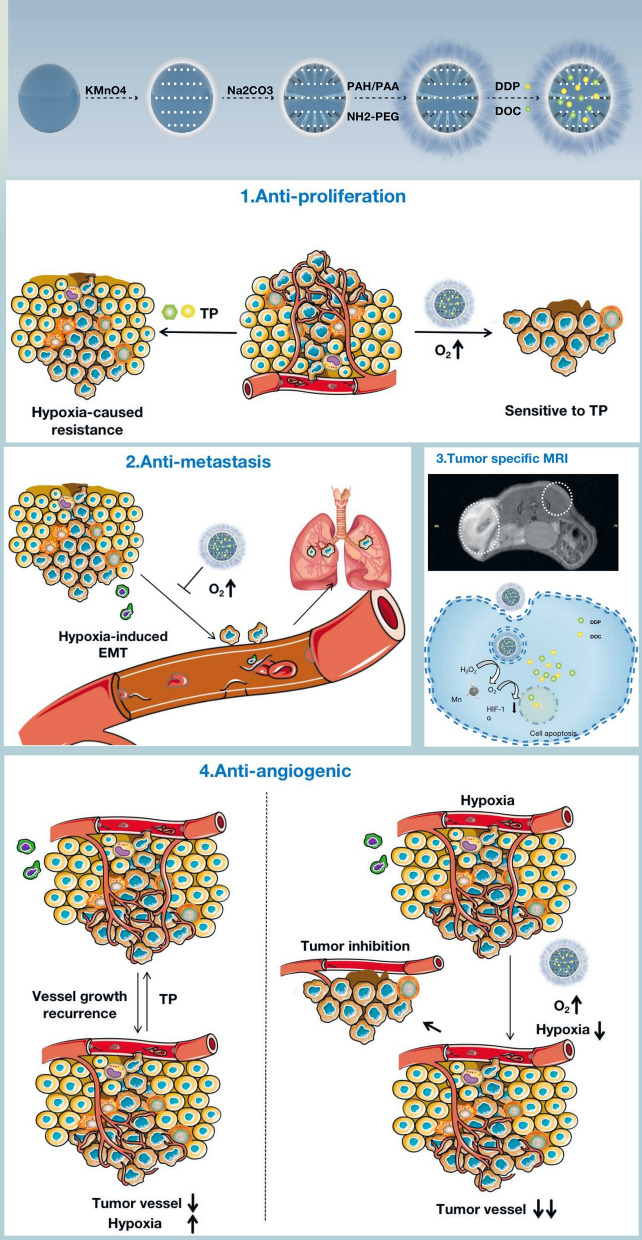

**Supplementary Information:**

The online version contains supplementary material available at 10.1186/s12951-021-00901-9.

## Highlights

- A theranostic platform has been developed using hollow mesoporous MnO_2_ (H-MnO_2_) nanoshells.
- H-MnO_2_ nanoshells trigger H_2_O_2_ decomposition into O_2_, reducing TME hypoxia and HIF-1α expression.
- H-MnO_2_ nanoshells exert an anti-angiogenic effect by downregulating HIF-1α expression.
- In combination with chemotherapy, H-MnO2 nanoshells have excellent efficacy in pulmonary metastasized oral squamous cell carcinoma.

## Introduction

The tumor microenvironment (TME) is characterized by oxygen-deficient solid tumors. Hypoxic conditions significantly promote tumor heterogeneity and increase metastatic spread [1–3]. Furthermore, undesirable angiogenesis and immunosuppression are caused by disordered metabolism and unstable genome phenotype accelerated in hypoxic TMEs, which consistently contribute to tumor resistance to various oxygen-related therapeutics [4, 5]. Many of the hallmarks of metastatic cancers are related to the hypoxic TME [6], which stimulates hypoxia-inducible factor (HIF)-driven transcriptional responses that upregulate the expression of hypoxia-inducible genes that facilitate invasion and metastasis [7, 8]. These hypoxic effects play essential roles in the outcomes of various cancer therapies [5, 9, 10].

Nanoscale drug delivery systems (nano-DDSs) have been regarded as an ideal approach to overcome the hypoxic TME, as nano-DDSs are capable of responding to the inherent acidic and hypoxic features of the TME [11, 12]. Recently, several studies have focused on the development of manganese dioxide (MnO_2_) nanostructures that can decompose under the acidic TME [13–16], generating Mn^2+^ ions that could enhance T1 images during magnetic resonance imaging (MRI) [17, 18]. Accordingly, MnO_2_ nanostructures could offer a safe DDS without long-term toxicity in in vivo therapy [13–16]. MnO_2_ nanostructures could also relieve tumor hypoxia by triggering the decomposition of H_2_O_2_ that is present in the TME [19, 20]. Hollow nanostructures with mesoporous shells (such as hollow mesoporous silica) have large cavities that have been demonstrated to be excellent drug loading/delivery systems, loading high quantities of therapeutic agents, whose release may be precisely controlled by tuning the shell structure or coating [21, 22].

The combination of docetaxel and cisplatin (TP) has become a first-line anticancer therapy in advanced OSCC, which provides good progression-free survival (PFS) and overall survival (OS) [23, 24]. Previous research has also demonstrated the limitations caused by TME hypoxia, which contributes to metastasis and angiogenesis of OSCC [25–28]. TP can be co-loaded into the H-MnO_2_-PEG nano-platform (H-MnO_2_-PEG/TP) with high loading capacities. Under acidic pH, the rapid decomposition of the MnO_2_ nanoshells leads to the release of the loaded drugs (TP), while simultaneously resulting in significantly enhanced T1-images during MRI.

Lung metastasis is a common feature of advanced OSCC and associated with a poor prognosis (5-year OS < 30%), which is contributed to by hypoxia-induced resistance [26, 27]—unlike pulmonary metastasis of HER2-positive breast cancer, which can be effectively treated through targeted therapies. Currently, there is a lack of effective targeted treatments for OSCC lung metastasis. Moreover, previous research has shown that HIF, which can be downregulated after hypoxia is relieved, is a promoting factor of angiogenesis. Considering MnO_2_ are able to target the TME and generate O_2_, we hypothesized that using hollow MnO_2_ nanostructures as smart DDSs may achieve an ideal therapeutic effect in the application of pulmonary metastasis of OSCC by reversing hypoxia-induced chemotherapy resistance.

Previous studies have designed similar smart DDSs to achieve tumor-specific enhanced combination therapy [29, 30]. In a previous system, mesoporous MnO_2_ shells combined with chlorine e6 (Ce6) and doxorubicin (DOX) were used to treat 4T1 cells in a subcutaneous model, which obtained an ideal effect. To the best of our knowledge, the therapeutic effect of MnO2 particles on OSCC pulmonary metastasis has not been assessed. In our research, we aimed to develop a system that could target OSCC lung metastasis by using pH-sensitive mesoporous MnO2 nanoshells co-loaded with first-line OSCC chemotherapeutic drugs, TP. We also assessed whether these TP-loaded MnO2 nanoshells were effective against TP-resistant OSCC cells.

## Materials and methods

### Materials

Tetraethyl orthosilicate (TEOS), poly(allylamine hydrochloride) (PAH, MW≈15,000), and polyacrylic acid (PAA, MW ≈ 1800) were purchased from Sigma-Aldrich (USA). Potassium permanganate (KMnO_4_) and sodium carbonate (Na_2_CO_3_) were obtained from Sinopharm Chemical Reagent CO., Ltd. (China). Coumarin-modified cisplatin and rhodamine-modified docetaxel were purchased from Xian Qiyue Biotechnic CO., Ltd. (China).

### Synthesis of H-MnO_2_-PEG/TP

Solid silica nanoparticles (sSiO_2_) were synthesized following the reported method [31]. Then, an aqueous solution of KMnO_4_ (300 mg) was added dropwise to the sSiO_2_ suspension (40 mg) under ultrasonication. After 6 h, the precipitate was obtained by centrifugating the suspension at 14,800 rpm. The as-prepared mesoporous MnO_2_-coated sSiO_2_ were dissolved in 2 M Na_2_CO_3_ aqueous solution at 60 °C for 12 h. The obtained hollow mesoporous MnO_2_ (H-MnO_2_) nanoshells were centrifuged and washed with water several times. Then, 5 ml of the H-MnO_2_ solution (2 mg/ml) was added to 10 ml PAH solution (5 mg/ml) under ultrasonication. After stirring for 2 h, the above solution was centrifuged and washed with water. The obtained H-MnO_2_/PAH solution was added drop wise to PAA (10 ml, 5 mg/ml) under ultrasonication. After 2 h of stirring, the above solution was centrifuged and washed with water, before it was mixed with mPEG-5 K-NH_2_ (50 mg) under ultrasonication for 30 min. After adding EDC (15 mg) and stirring for 12 h, the prepared H-MnO_2_-PEG was collected by centrifugation and washed with water three times. For docetaxel and cisplatin (TP) loading, the H-MnO_2_-PEG solution (0.2 mg/ml) was mixed with different concentrations of TP for 12 h. TP were co-loaded into H-MnO_2_-PEG with appropriate concentrations, yielding H-MnO_2_-PEG/TP which were used in further experiments.

### Characterization

Scanning electron microscopy (SEM; JSM-2100F, JEOL, Tokyo, Japan) was applied to characterize the nanoparticle morphology. Ultraviolet–visible (UV–Vis) spectra were measured with a PerkinElmer Lambda 750 UV/Vis/NIR spectrophotometer. Nanoparticle size and zeta potential were determined by a Malvern Zetasizer (ZEN3690, Malvern, UK) and Nano ZS90 (Malvern, UK). Surface area and pore size were measured by Surface Area and Porosity Analyzer (Micromeritics Instrument Corp. ASAP2050). The functional groups and chemical structure of the nanofibers were performed by Fourier transform infrared (FT-IR) spectroscopy (Nicolet iS50) in the wavenumber range of 4000–400 cm^–1^.

### Degradation and drug release studies

H-MnO_2_-PEG was incubated with PBS at different pH values (4.5, 5.5, 6.5, and 7.4) for different time periods (0–36 h). At a given time point, the solution was measured by SEM and UV–Vis spectrometry for characterization. To study the TP release, a solution of H-MnO_2_-PEG/TP was dialyzed against PBS with different pH values (4.5, 5.5, 6.5, and 7.4) at room temperature. The amount of TP released at different time points was measured by UV–Vis spectrometry.

### In vitro cell experiments

The tongue squamous cell carcinoma cell line CAL27 was purchased from ATCC (Manassas, VA, USA). Mice oral squamous cell carcinoma cell line SCC7 was provided as a gift from the Nanjing Medical University (China). For cell toxicity assays, cells were seeded into 96-well plates (1 × 10^4^ per well) for 24 h and incubated with a series of concentrations of TP. 3-(4,5-dimethylthiazol-2-yl)-2,5-diphenyltetrazolium bromide (MTT) solution (0.5 mg/ml) was added to the wells to measure the cell viability of the treated cells relative to the untreated cells. For confocal fluorescence microscopy, CAL27 cells were seeded onto coverslips at the bottom of a dish containing H-MnO_2_-PEG/TP (docetaxel: 3.1 nM, cisplatin: 18 nM) for different incubation times (1, 4, 8, and 12 h). After washing with PBS three times, the cells were labeled with 4′,6-diamidino-2-phenylindole (DAPI) and imaged using a laser scanning confocal microscope (Leica SP5). Lentivirus-transduced stable cells were seeded into 6-well plates at a density of 1000 cells per well and incubated for 10–14 days. The colonies were fixed and stained, and those with more than 50 cells were counted under a dissecting microscope.

### Western blot analysis

Proteins were extracted from OSCC cells treated with H-MnO_2_-PEG/TP (docetaxel: 3.1 nM, cisplatin: 18 nM) for 0, 2, 4, 8, 12, and 16 h. The membranes were blocked by adding QuickBlock™ Blocking Buffer at room temperature for 20 min and then incubating with primary antibodies (β-actin, 1:1000; HIF-1α, 1:1000) overnight at 4 °C. The membranes were then incubated with fluorescently-labelled anti-rabbit IgG secondary antibodies (7704, Cell Signaling Technology, USA) at a 1:10000 dilution for 1 h at room temperature. Immunoreactive bands were detected using enhanced chemiluminescence. The observation and analysis of immunoreactive bands were performed using the Odyssey Infrared Imaging System (LI-COR Biosciences, USA).

### Animal models

SPF BALB/c nude mice (nu/nu, 4 weeks old, weighing approximately 20 g) were purchased from Shanghai experimental animal center (Shanghai, China) and placed in the SPF facility of the Ninth People's Hospital, Shanghai Jiao Tong University School of Medicine. All laboratory procedures were approved by the Laboratory Animal Care and Use Committee at the hospital. High metastatic oral squamous cell carcinoma was established. After luciferase lentivirus transfection, CAL27 cells (1 × 10^6^) were suspended in 50 μl of PBS and injected by intravenous injection (IV) or subcutaneous injection (SC). The mice bearing CAL27 tumors were treated 10 days after injection.

### In vivo imaging

In vivo fluorescence imaging was performed using the Maestro In-Vivo Fluorescence Imaging System (CRi Inc., USA). MRI was conducted under a BioSpec 70/20 USR (Bruker, USA) with a special coil for small animal imaging.

### Immunohistochemistry

CAL27 tumor-bearing mice were injected intravenously with PBS or H-MnO_2_-PEG/TP. Liver, kidney, and lungs bearing tumors were surgically excised 20- or 120-min post injection. Tissue sections (4 mm) were stained with hematoxylin and eosin (HE). Terminal deoxynucleotide transferase dUTP notch end labeling (TUNEL) was used to detect apoptotic cells. To detect oxidation, the tumor sections were treated with mouse anti-HIF1α primary antibody (dilution 1:200, Abcam Inc. USA) and Alexa Flour^®^ 488-conjugated goat anti-rabbit secondary antibody (dilution 1:200, CST Inc. USA) following the instructions. Tumor blood vessels were stained by anti-CD31 mouse monoclonal antibody (dilution 1:200, Abcam Inc.) and Alexa Flour^®^ 555-conjugated goat anti-mouse secondary antibody (dilution 1:200, CST Inc., USA), subsequently. Cell nuclei were stained with DAPI (dilution 1:5000, Invitrogen, USA). The obtained slices were observed by confocal microscopy (Leica SP5, Germany).

### In vivo cancer treatment

CAL27 tumor-bearing mice were injected via i.p. with 100 μl of PBS, TP, or H-MnO_2_-PEG/TP (dose of MnO_2_: 10 mg/kg, docetaxel: 10 mg/kg, cisplatin: 2.5 mg/kg). Body weights were monitored every 2 days for 4 weeks. Tumor progression was monitored using Maestro In-Vivo Fluorescence Imaging System every 2 weeks. The tissue and tumor slices were stained by HE, following standard protocol.

## Results

### Synthesis and characterization of H-MnO_2_-PEG

**Fig. 1:**
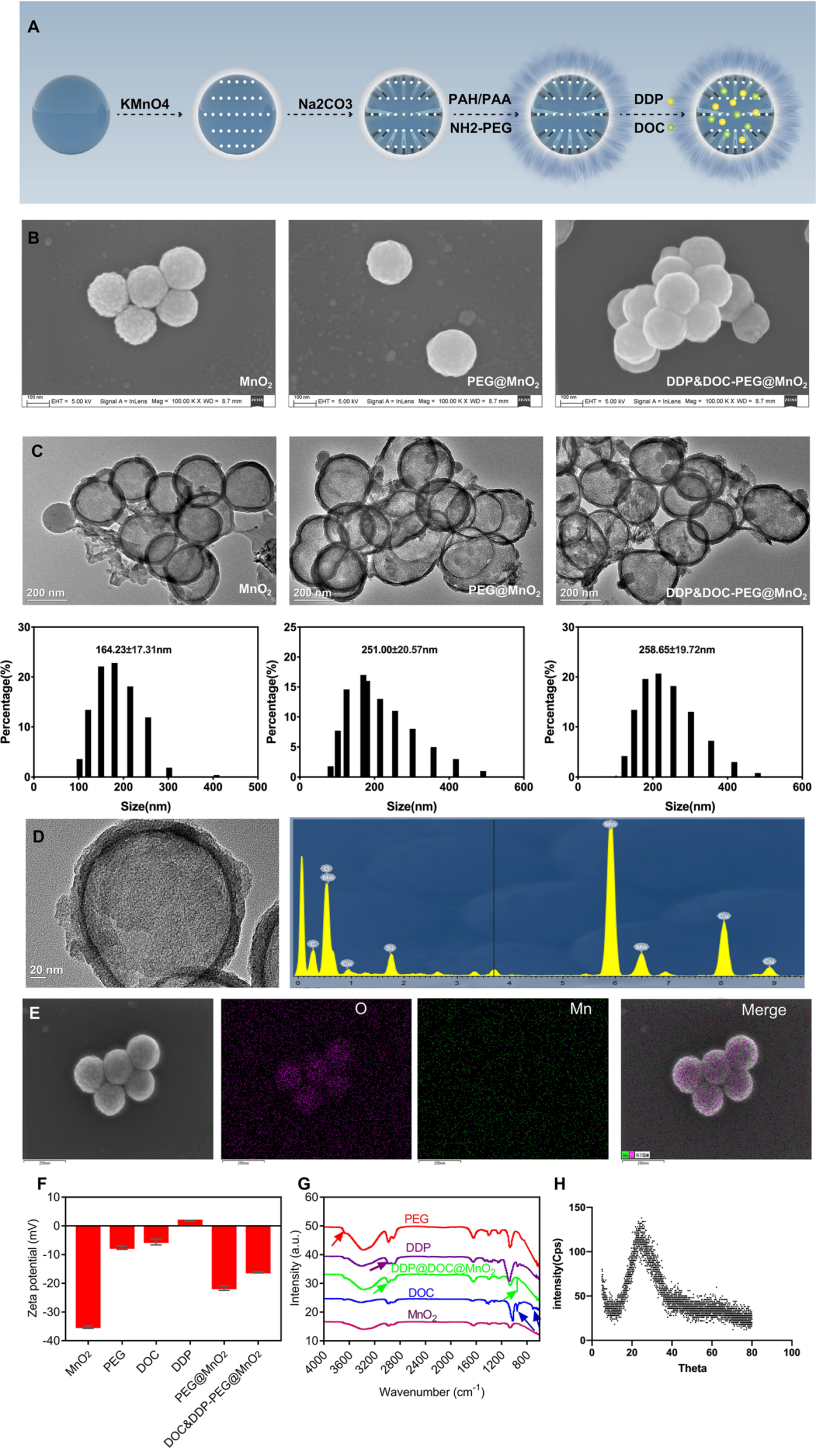
Synthesis and characterization of H-MnO_2_-PEG nanoshells: **a** step-by-step synthetic scheme of H-MnO_2_-PEG nanoparticles and subsequent dual-drug loading; **b** SEM images of MnO_2_, PEG@MnO_2_ and H-MnO_2_-PEG/TP; **c** TEM images of MnO_2_, PEG@MnO_2_ and H-MnO_2_-PEG/TP; **d** HRTEM for H-MnO_2_-PEG nanoshells; **e** SEM–EDS imaging and elemental mapping for H-MnO_2_-PEG nanoshells; **f** zeta potentials of nanoparticles obtained at different steps of fabrication; **g** FT-IR of free PEG, DDP, DDP@DOC@MnO_2_, DOC and MnO_2_; **h** XRD for H-MnO_2_-PEG

The H-MnO_2_-PEG/TP synthetic process is illustrated in Fig. 1a. Monodispersed silica nanoparticles were synthesized by hydrolyzation of tetraethyl orthosilicate (TEOS) and then used immediately as the hard template. A uniform layer of mesoporous MnO_2_ was grown on the surface of the as-made silica nanoparticles by mixing them with KMnO_4_, which was reduced by unreacted organosilica existing on the prepared silica nanoparticles. The H-MnO_2_ nanoshells were obtained after incubating MnO_2_@SiO_2_ nanoparticles with a Na_2_CO_3_ solution to dissolve silica. To enhance their water solubility and physiological stability, H-MnO_2_ nanoshells were modified with PEG through a layer-by-layer (LBL) polymer-coating method. In this process, negatively-charged H-MnO_2_ nanoshells were coated with a cationic polymer PAH and then an anionic polymer PAA through electrostatic interaction. Amino-terminated PEG (NH_2_-PEG) was then conjugated to the surface of PAA-coated H-MnO_2_ nanoshells via amide formation, producing H-MnO_2_-PEG nanoshells. TP were simultaneously loaded into the hollow structure of the H-MnO_2_-PEG nanoshells, yielding H-MnO_2_-PEG/TP. SEM and TEM images revealed the spherical morphology and the hollow structure of the H-MnO_2_-PEG nanoshells (Fig. 1b, c). The hollow structure of the H-MnO_2_-PEG nanoshells was further confirmed by SEM-EDS (Fig. 1d, e). The change in zeta potential for the nanoparticles obtained at different steps of synthesis are shown in Fig. 1f. In the process of surface functionalization, the step wise altered spectrogram indicated successful LBL coating of polymers on the nanoparticles (Fig. 1g). No diffraction phenomena was observed according to XRD result (Fig. 1h), which indicates a low crystallinity structure of synthesized nanoparticles. Because of the structural defects, oxygen vacancies and low manganese of the low crystallinity structure synthesized at low temperature, it is easier to degradate in vivo [32, 33].

### pH-dependent nanoparticle decomposition and drug behavior

**Fig. 2:**
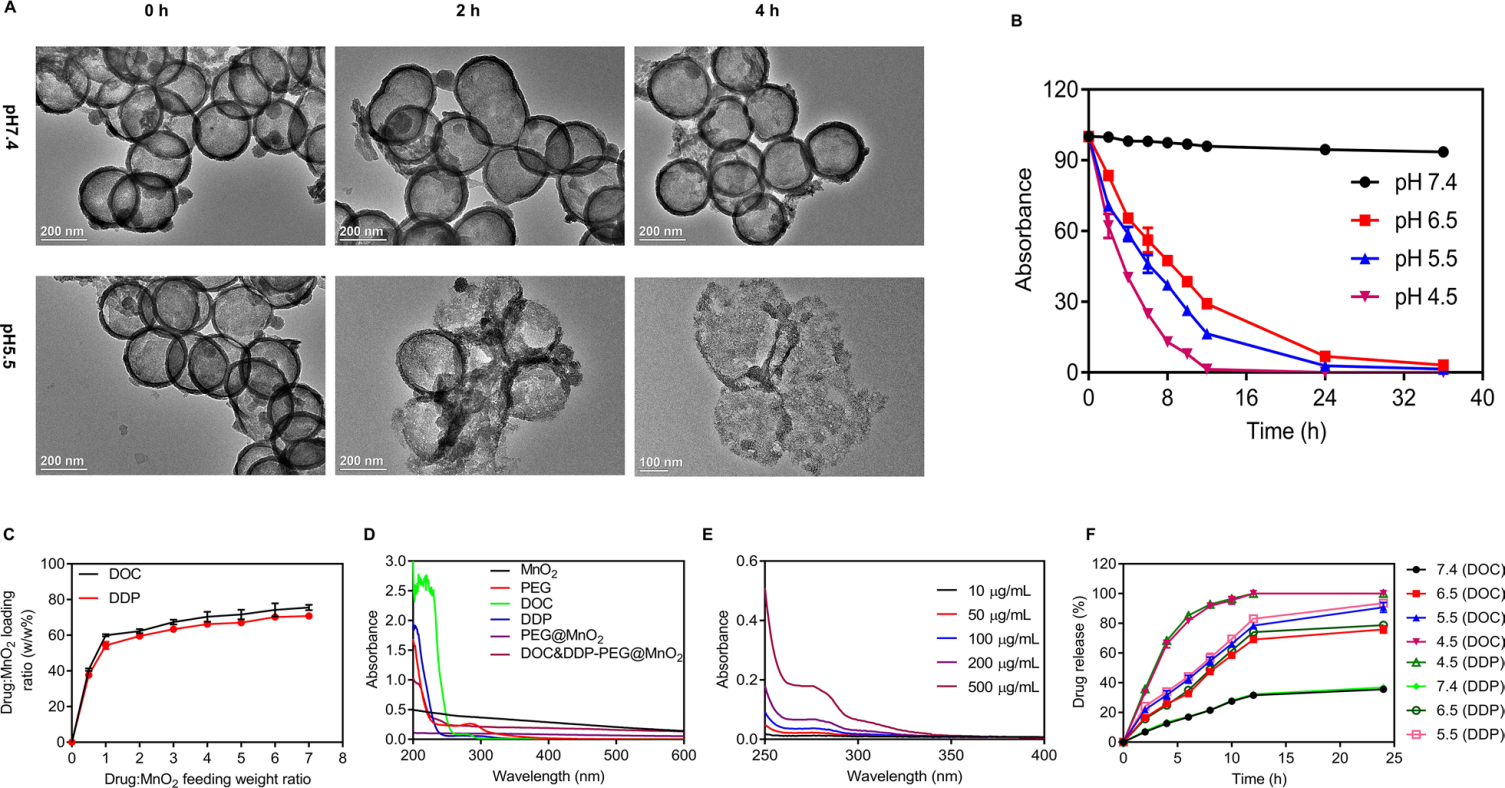
pH-dependent nanoparticle decomposition and drug behaviors of H-MnO_2_-PEG/TP nanoshells: **a** TEM images of H-MnO_2_-PEG nanoshells after incubation in buffers with different pH values (7.4 and 5.5) for various periods of time; **b** degradative behavior of H-MnO_2_-PEG nanoshells dispersed in solutions with different pH values (7.4, 6.5, 5.5, and 4.5) determined by the absorbance of MnO_2_; **c** DOC- and DDP-loading weight ratios in H-MnO_2_-PEG nanoshells at different drug to MnO_2_ ratios; **d** UV–Vis spectra of free PEG, MnO_2_, DOC, DDP, H-MnO_2_-PEG, and H-MnO_2_-PEG/TP nanoshells; **e** UV–Vis spectra of H-MnO_2_-PEG/TP nanoshells measuring drug release with increasing concentration; and **f** percentages of released DOC and DDP from H-MnO_2_-PEG/TP nanoshells over time in the presence of 10% fetal bovine serum (FBS) at different pH values (7.4, 6.5, 5.5, and 4.5). Data are presented as means ± standard deviation (s.d.) (n = 3)

It is well known that MnO_2_ is stable at neutral and alkaline pH but can decompose into Mn^2+^ at reduced pH [34]. Therefore, TEM images of H-MnO_2_-PEG incubated in PBS with different pH values (5.5 and 7.4) at different processing times were recorded (Fig. 2a). The morphology of H-MnO_2_-PEG nanocrystals showed no significant change at pH 7.4 after eight hours, indicating that H-MnO_2_-PEG nanocrystals were stable in neutral environments. However, due to the decomposition of MnO_2_ into Mn^2+^ ions, H-MnO_2_-PEG showed time-dependent degradation behavior in acidic solutions. The degradation rate was determined by decreasing the MnO_2_-characteristic absorption band (Fig. 2b), which appears to be stable at pH 7.4, but rapidly decreases at pH 6.5, 5.5, and 4.5, further demonstrating the ultra-sensitive pH-responsive degradation behavior of H-MnO_2_-PEG.

The H-MnO_2_-PEG with mesoporous shells were expected to have an efficient drug-loading ability. H-MnO2-PEG nanoshells were loaded with TP. Under ultrasonication, H-MnO_2_-PEG nanoshells were incubated and stirred with different concentrations of free DOC and DDP. Drug-loading capacities was evaluated by UV–Vis spectroscopy. At the feeding weight ratio of 1:1 (DOC:DDP), the drug loading capacity of the nanoshells was high: 75.53% (DOC:MnO_2_) and 71.75% (DDP:MnO_2_; Fig. 2c). DOC and DDP could also be simultaneously loaded into the hollow structure of H-MnO_2_-PEG nanoshells, obtaining dual drug co-loaded H-MnO_2_-PEG/TP nanoparticles (Fig. 2d) and enhanced release with increasing concentration (Fig. 2e). Drug-release behaviors of DOC and DDP from H-MnO_2_-PEG/TP were studied in solutions with different pH values (Fig. 2f). Compared to the slow drug-release profiles of H-MnO_2_-PEG/TP at pH 7.4, the release speeds of both DOC and DDP were found to be much faster in mild acidic solutions at pH 6.5, 5.5, and 4.5, owing to the acidic-triggered decomposition of H-MnO_2_ nanocarriers into Mn^2+^ ions.

### In vitro experiments with H-MnO_2_-PEG/TP

**Fig. 3:**
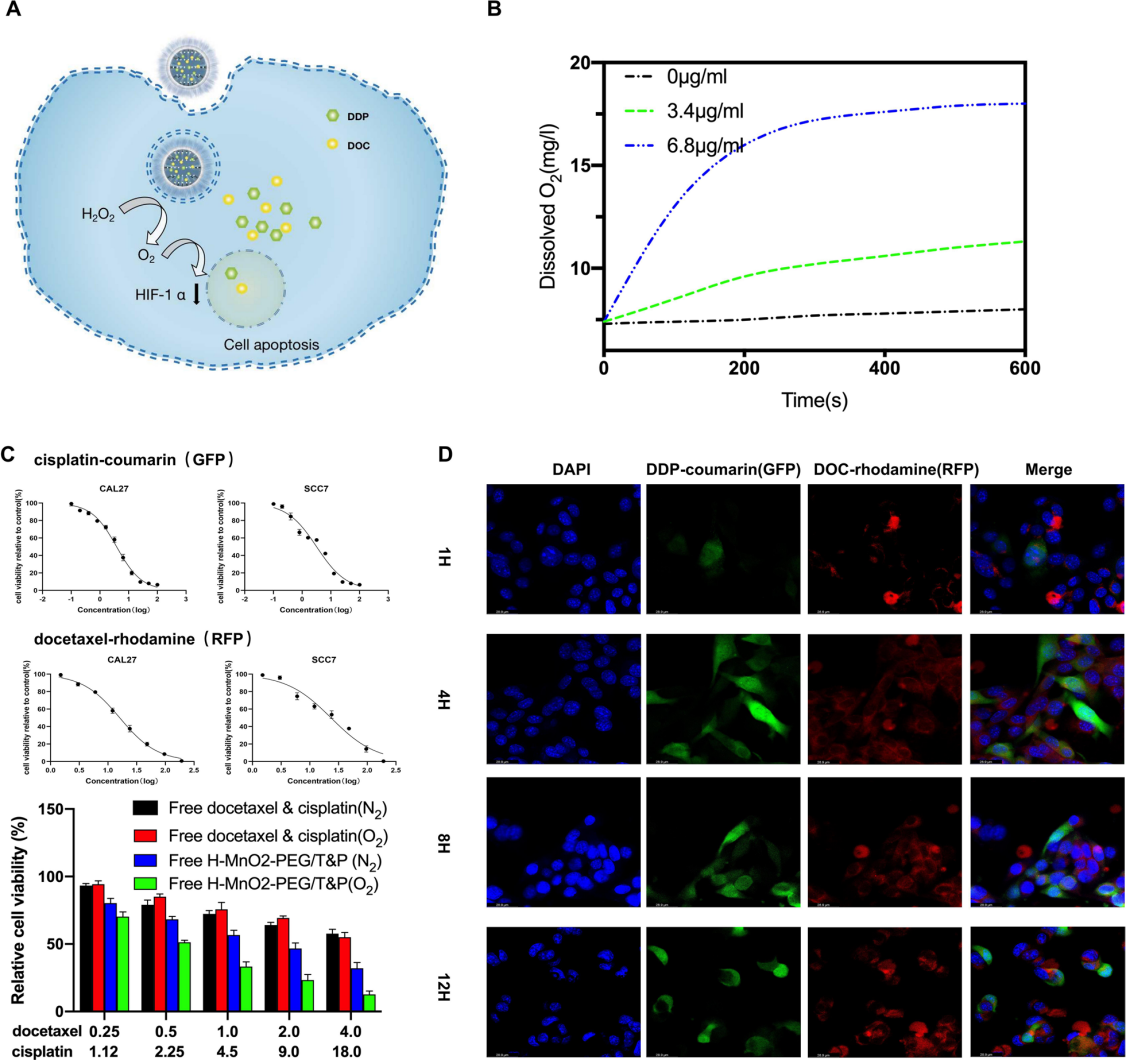
In vitro experiments with H-MnO_2_-PEG/TP nanoshells: **a** a scheme illustrating H-MnO_2_-PEG/TP nanoshells in pH-responsive drug delivery; **b** the changes in O_2_ concentration in H_2_O_2_ solutions (100 μM) after various concentrations of H-MnO_2_-PEG/TP nanoshells were added; **c** after the treatment of different concentrations of DDP/DOC within 24 h, cell viability was assessed using an MTT assay where IC50 of DDP/DOC was measured in SCC7 and CAL27 cells (in vitro TP treatment was measured with and without H-MnO_2_-PEG nanoshells in N_2_ or O_2_ atmospheres in CAL27 cells); and **d** confocal microscopy images of CAL27 cells treated with H-MnO_2_-PEG/TP at different times points. Blue, green, and red represent DAPI, DDP, and DOC fluorescence, respectively. Date are presented as means ± s.d. (n = 5)

**Fig. 4:**
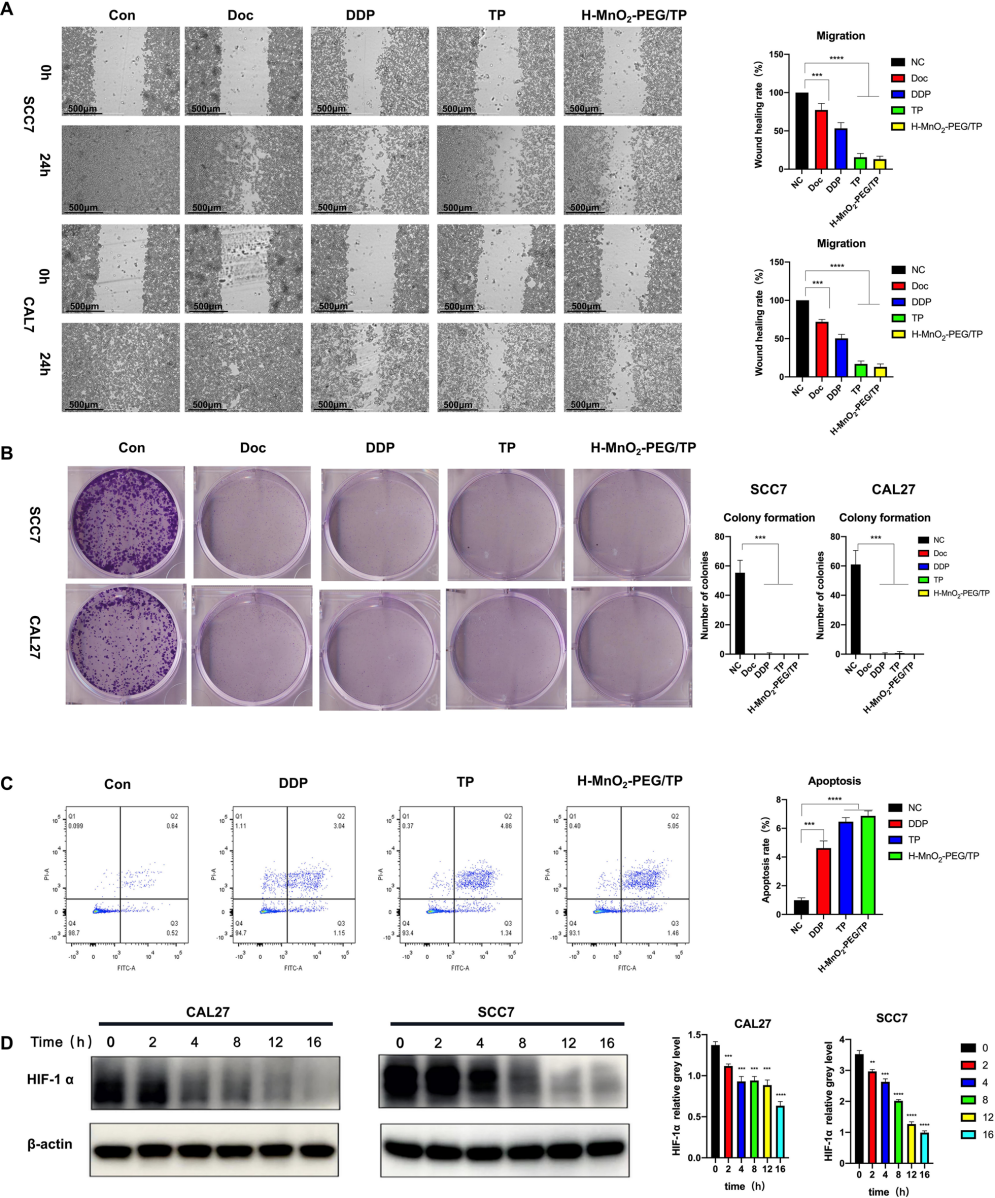
Antitumor function of H-MnO_2_-PEG/TP nanoshells in vitro: **a** Wound healing was performed with SCC7 and CAL27 cells after treatment with PBS, DOC, DDP, TP, and H-MnO_2_-PEG/TP nanoshells; **b** colony formation assays were performed with SCC7 and CAL27 cells after treatment with PBS, DOC, DDP, TP, and H-MnO_2_-PEG/TP nanoshells; **c** CAL27 cells were treated with indicated concentrations PBS, DDP, TP, and H-MnO_2_-PEG/TP nanoshells for 48 h before staining with Annexin V and propidium iodide (PI), and the apoptotic rates were determined by flow cytometry; and **d** Western blotting showed decreased HIF-1α after treatment with indicated concentrations of H-MnO_2_-PEG/TP nanoshells for 48 h in the CAL27 and SCC7 cells

As described in previous research, the efficacy of TP is limited by the hypoxic TME of a solid tumor [35–39]. Considering the presence and concentration of endogenous H_2_O_2_ in most types of solid tumors is in the range of 100 μM [40], we then tested the ability of H-MnO_2_ to act as a catalyst and induce the decomposition of H_2_O_2_. An oxygen probe was used to measure the oxygen released into the solution after adding H_2_O_2_ (100 μM) with different concentrations of H-MnO_2_-PEG nanoshells. Without the addition of H-MnO_2_-PEG nanoshells, the dissolved O_2_ in the H_2_O_2_ solution was maintained at a low and stable level. H-MnO_2_-PEG nanoshells can effectively trigger the rapid generation of O_2_ from H_2_O_2_ in a MnO_2_ concentration-dependent manner (Fig. 3b). Then, the in vitro efficacy of H-MnO_2_-PEG nanoshells as a multifunctional DDS was assessed by using CAL27 cells. As expected, no significant difference was observed in OSCC cells that were treated in different concentrations of H-MnO_2_-PEG (Additional file 1: Figure S1). Then, SCC7 and CAL27 cells were used to determine the IC50 of DOC and DDP. Cells were then treated with H-MnO_2_-PEG/TP in either an N_2_ or O_2_ environment, and the cell viabilities were determined by an MTT assay after incubation for 24 h (Fig. 3c). We used H-MnO_2_-PEG/TP for in vitro combination treatment. CAL27 cells incubated with H-MnO_2_-PEG/TP for different periods of time were then imaged by a confocal fluorescence microscope (Fig. 3d). Both DDP-coumarin (GFP) and DOC-rhodamine (RFP) fluorescence inside cells was significantly enhanced with increased incubation time. The colony-formation and -migration abilities were significantly decreased in CAL27 and SCC7 cells in the H-MnO_2_-PEG/TP group compared to the control group (p < 0.01) (Fig. 4a, b). Flow cytometry assays also confirmed that apoptosis was induced by H-MnO_2_-PEG/TP (Fig. 4c). Hypoxia is a key concern during the treatment of non-small cell lung cancer (NSCLC) [39], as with pulmonary metastasis of OSCC, and hypoxia-inducible factor 1 alpha (HIF-1α) has been associated with increased tumor resistance to therapeutic agents such as cisplatin. To further evaluate, the downregulation of HIF-1α induced by H-MnO_2_-PEG/TP was confirmed using western blot. (Fig. 4d).

### In vivo and ex vivo imaging with H-MnO_2_-PEG/TP

**Fig. 5:**
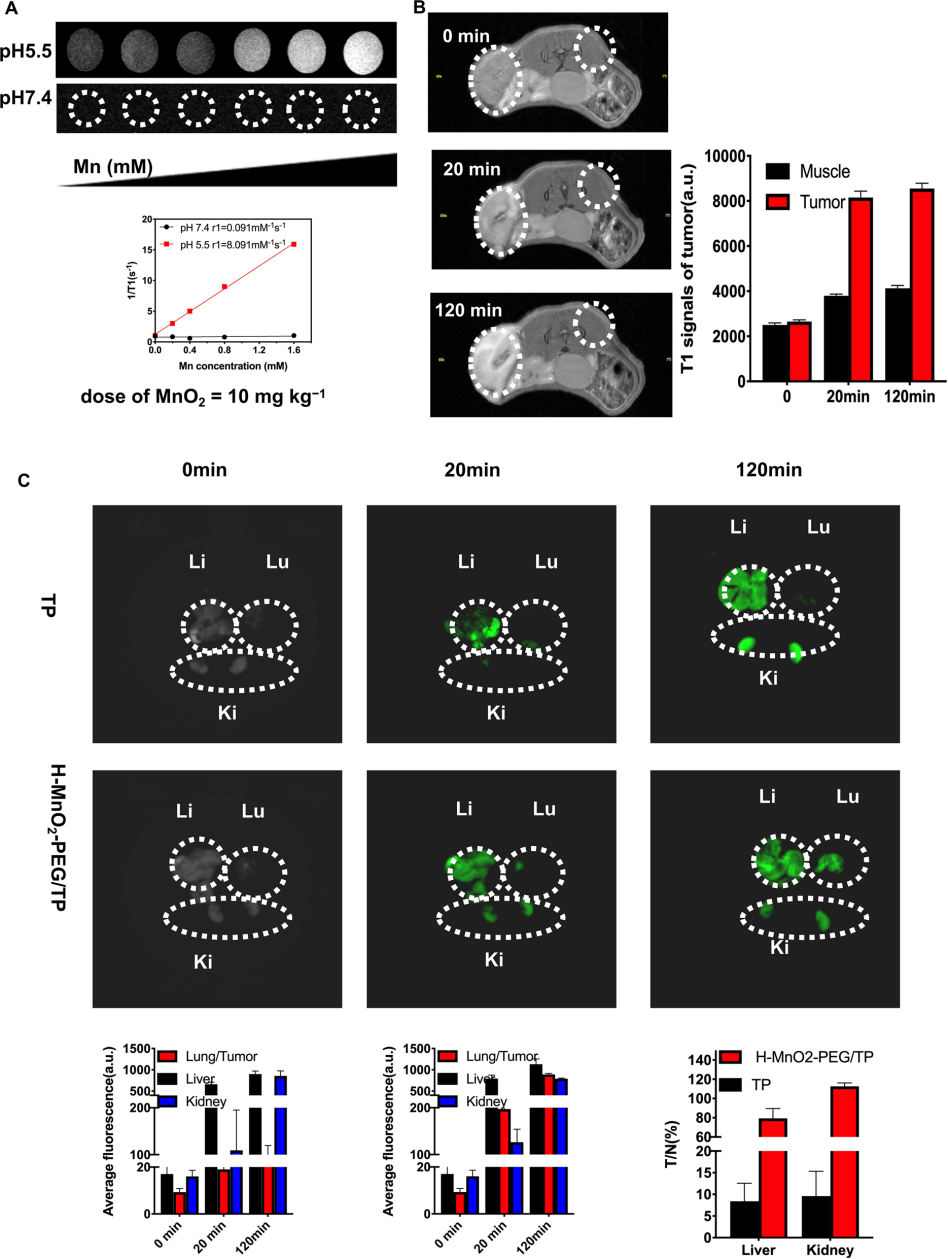
In vivo and ex vivo imaging with H-MnO_2_-PEG/TP nanoshells: **a** images from T1-weighted MRI of the H-MnO_2_-PEG/TP nanoshells recorded using BioSpec 70/20 USR at different pH values (5.5 and 7.4). The transverse relativities (r1) were 8.091 and 0.091 mM^−1^ s^−1^ for H-MnO_2_-PEG/TP nanoshells at pH 5.5 and 7.4, respectively. **b** Images from T1-MRI of CAL27 tumor-bearing mice before and after local injection of H-MnO_2_-PEG/TP nanoshells within normal and tumor subcutaneous tissues (three mice per group). Quantified T1-MR signals in muscle and tumor before and after injection with H-MnO_2_-PEG/TP nanoshells. **c** Ex vivo fluorescence images of major organs and pulmonary metastasis tumors in Balb/c mice dissected from mice 2 h after injection (Ki, Lu and Li stands for kidney, lung, and liver, respectively). Semi-quantitative analysis of ex vivo fluorescence images in different organs. Data are presented as means ± s.d. (n = 3 mice per group)

After confirmation of in vitro efficacy of H-MnO_2_-PEG/TP nanoshells, the effect of H-MnO_2_-PEG/TP in an OSCC subcutaneous bearing or pulmonary metastasis model was assessed. Mn^2+^ ions with five unpaired 3d electrons could decompose from MnO_2_ under the acidic conditions of the TME and is known as a T1-shortening agent in MRI [41]. In solutions at different pH (5.5 and 7.4), the H-MnO_2_-PEG/TP incubated in solutions at pH 5.5 showed a brighter T1-shortening image compared with the image at pH 7.4 (Fig. 5a). To demonstrate the use of H-MnO_2_ nanoshells for tumor-specific imaging, H-MnO_2_-PEG/TP nanoshells were injected into tumor, and into muscle on the opposite side of the tumor in tumor-bearing mice for MRI (Fig. 5b). Caused by the acidic TME, the tumor showed significantly enhanced images in T1 signaling after injection of H-MnO_2_-PEG/TP nanoshells, whereas the muscle area with the same concentration of injected nanoparticles had reduced T1-signal enhancement (Fig. 5b). This phenomenon provides direct evidence that H-MnO_2_ has ultra-sensitive pH-responsive T1-MR contrast performance, which is particularly useful for tumor-specific imaging. After intravenous injection of H-MnO_2_-PEG/TP nanoshells (dose of MnO_2_: 10 mg/kg, docetaxel: 10 mg/kg, cisplatin: 2.5 mg/kg), in vivo fluorescence imaging was used to track the nanoparticles in CAL27 pulmonary metastasis Balb/c mice (Additional file 2: Figure S2). Semi-quantitative biodistribution data based on ex vivo imaging of major organs and tumors was collected two hours post injection, indicating a high tumor uptake of H-MnO_2_-PEG/TP (Fig. 5c). Notably, strong fluorescence was found in the kidneys of mice after H-MnO2-PEG/TP injection, illustrating a rapid renal clearance of the decomposed nanoshells. Previous research [42] used the tumor to normal organ uptake ratio (T/N ratio) to evaluate tumor targeting efficiency. Tumor uptake of TP alone (without the nanoparticle) was extremely low, indicating a low T/N ratio. Compared to the TP group, the T/N ratio increased significantly in mice treated with H-MnO2-PEG/TP.

### In vivo chemotherapy treatment with H-MnO_2_-PEG/TP nanoshells

**Fig. 6:**
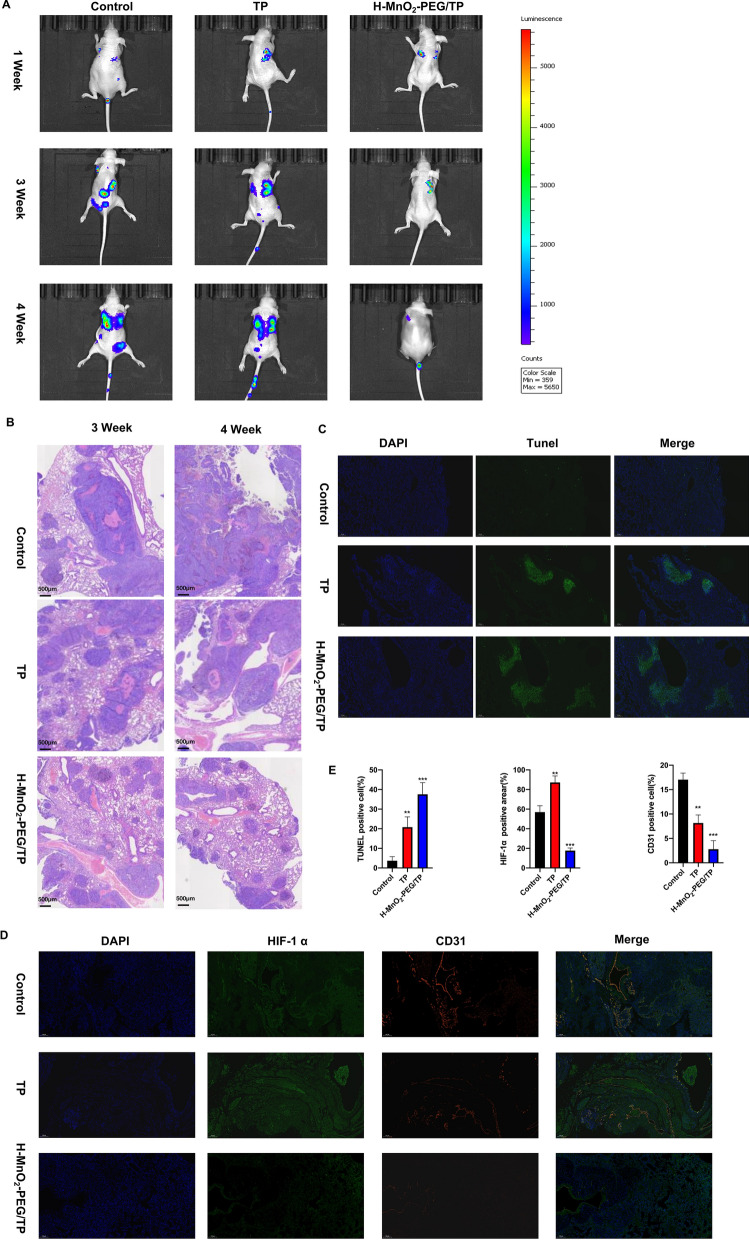
In vivo combination chemotherapy of H-MnO_2_-PEG/TP nanoshells. **a** In vivo fluorescence images of mice with CAL27 pulmonary metastasis taken at different time points (six mice per group). **b** HE-stained tumor slices collected from mice after the various treatments indicated. **c** The percentage of TUNEL-positive cells were assessed in formalin-fixed paraffin embedded sections from tumors in each group. **d** The nuclei, blood vessels, and hypoxic areas stained with DAPI (blue), anti-HIF-1α antibody (green), and anti-CD31 antibody (red), respectively (three mice per group). Quantification of hypoxia and CD31 areas in tumors at different time points post injection of H-MnO_2_-PEG/TP nanoparticles. p values in (**e**) were calculated using a student *t*-test (***p < 0.001, **p < 0.01, *p < 0.05)

According to previous research [14, 30, 43], H-MnO_2_-PEG nanoparticles effectively increase lung metabolism. In vivo fluorescence imaging was used to evaluate tumor progression (Fig. 6a). At day 28, the tumors of all mice were collected. For tumors in mice treated with H-MnO_2_-PEG/TP nanoshells, their growth was partially delayed. Furthermore, HE staining of tumor slices showed that the majority of tumor cells were severely damaged in the H-MnO_2_-PEG/TP-treated group (Fig. 6b). To evaluate the biosafety of H-MnO_2_-PEG/TP nanoshells, HE staining of kidney and liver slices were obtained 4 weeks after drug application (Additional file 3: Figure S3). Compared to the control group, no obvious cell fibrosis, necrosis, or hypertrophy was observed in the H-MnO_2_-PEG/TP-treated group.

Terminal deoxynucleotide transferase dUTP notch end labeling (TUNEL) was used to detect apoptotic cells. H-MnO_2_-PEG/TP nanoshells upregulated apoptosis in vivo (Fig. 6c). It is known that tumor cells are able to constitutively produce H_2_O_2_, whose level has been reported to be in the range of 10–100 μM in many types of solid tumors [40]. Therefore, H-MnO_2_-PEG/TP nanoparticles may be able to trigger the decomposition of H_2_O_2_ generated by cancer cells, producing O_2_ in situ to relieve tumor hypoxia. The cell nuclei, blood vessels, and hypoxic areas were stained with 2-(4-amidinophenyl)-6-indolecarbamidine dihydrochloride (DAPI, blue), anti-CD31 antibody (red), and anti-HIF-1α antibody (green), respectively. Tumor slices collected at different time points in the H-MnO_2_-PEG/TP-treated group showed a reduction in green fluorescence compared to the control group, indicating that MnO_2_ accumulation in the tumor triggers H_2_O_2_ decomposition into O_2_, reducing tumor hypoxia (Fig. 6d).

## Conclusion

In summary, H-MnO_2_-PEG/TP nanoshells act as a multifunctional theranostic platform, responding to and modulating TME and suppressing OSCC pulmonary metastasis by overcoming chemotherapy resistance. The ultrasensitive pH responsiveness of H-MnO_2_-PEG/TP enables tumor-specific MRI and efficient drug release in acidic TMEs. The relieved tumor hypoxia by MnO_2_-triggered decomposition of endogenous tumor H_2_O_2_ offers remarkable benefits. HIF-1α expression was significantly reduced in vitro and in vivo, which relieves hypoxia and attenuates angiogenesis. Phenomena mentioned before could synergize the efficacy of chemotherapy and reverse angiogenesis in the TME to favor anticancer treatment. With its inherent biodegradability, this H-MnO_2_-based theranostic nanoplatform may find significant potential in clinical translation by providing a method of combining chemotherapy with antiangiogenesis therapy.

## Supplementary Information

**Additional file 1: Figure S1.** Cell viability of OSCC under different concentrations of H-MnO_2_-PEG.

**Additional file 2: Figure S2.** The in vivo fluorescence imaging of H-MnO_2_-PEG/TP in pulmonary metastasis Balb/c mice.

**Additional file 3: Figure S3.** HE staining of kidney and liver slices.

## Data Availability

Not applicable.
