# Supplementary figures and images for: Overcoming chemotherapy resistance using pH-sensitive hollow MnO2 nanoshells that target the hypoxic tumor microenvironment of metastasized oral squamous cell carcinoma
Source: J Nanobiotechnology. 2021 May 26;19:157. doi: 10.1186/s12951-021-00901-9 (PMC8157461; doi:10.1186/s12951-021-00901-9)

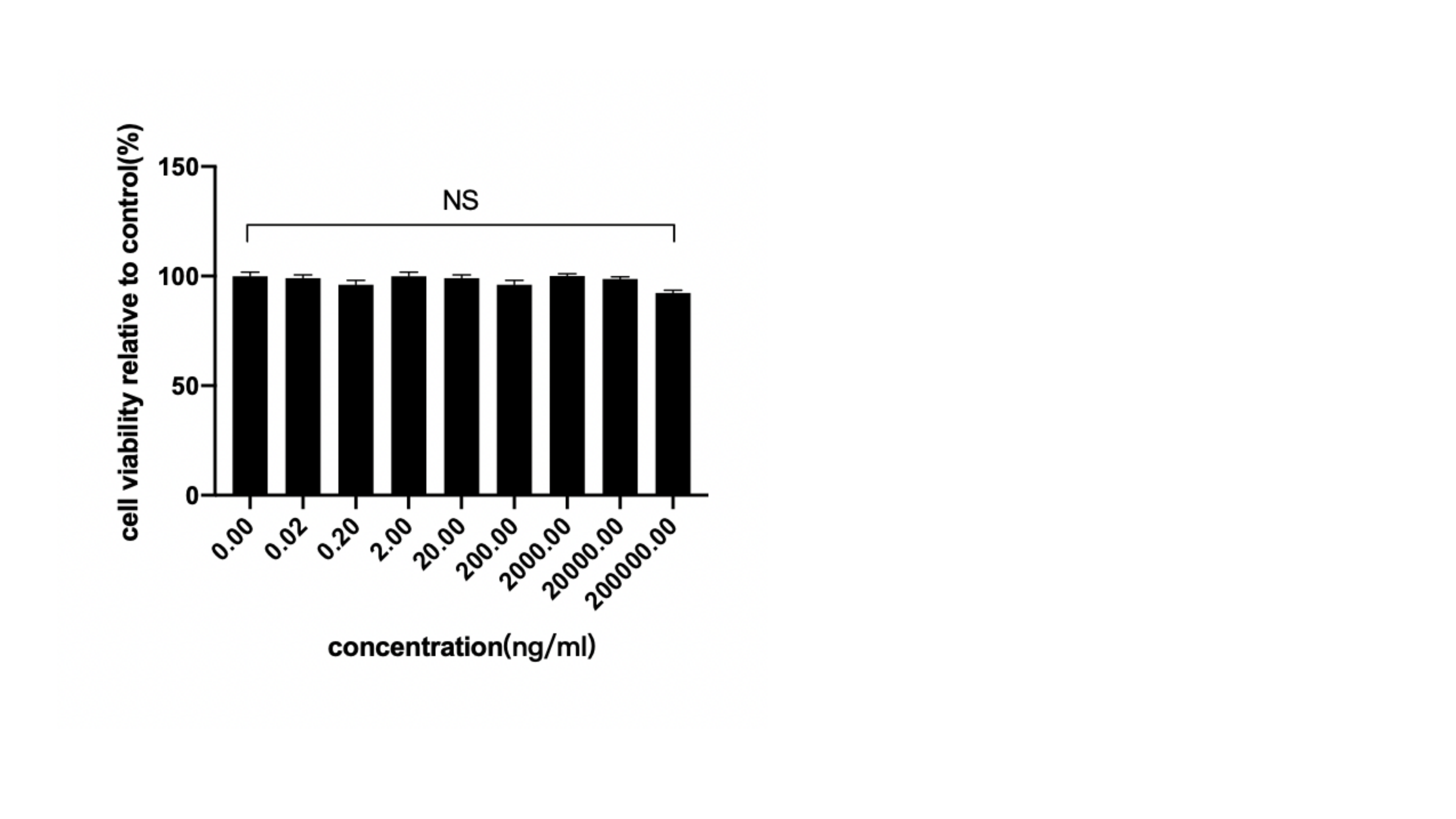

Supplement: Supplementary file 1 — Additional file 1: Figure S1. Cell viability of OSCC under different concentrations of H-MnO2-PEG. [file 12951_2021_901_MOESM1_ESM.tif]

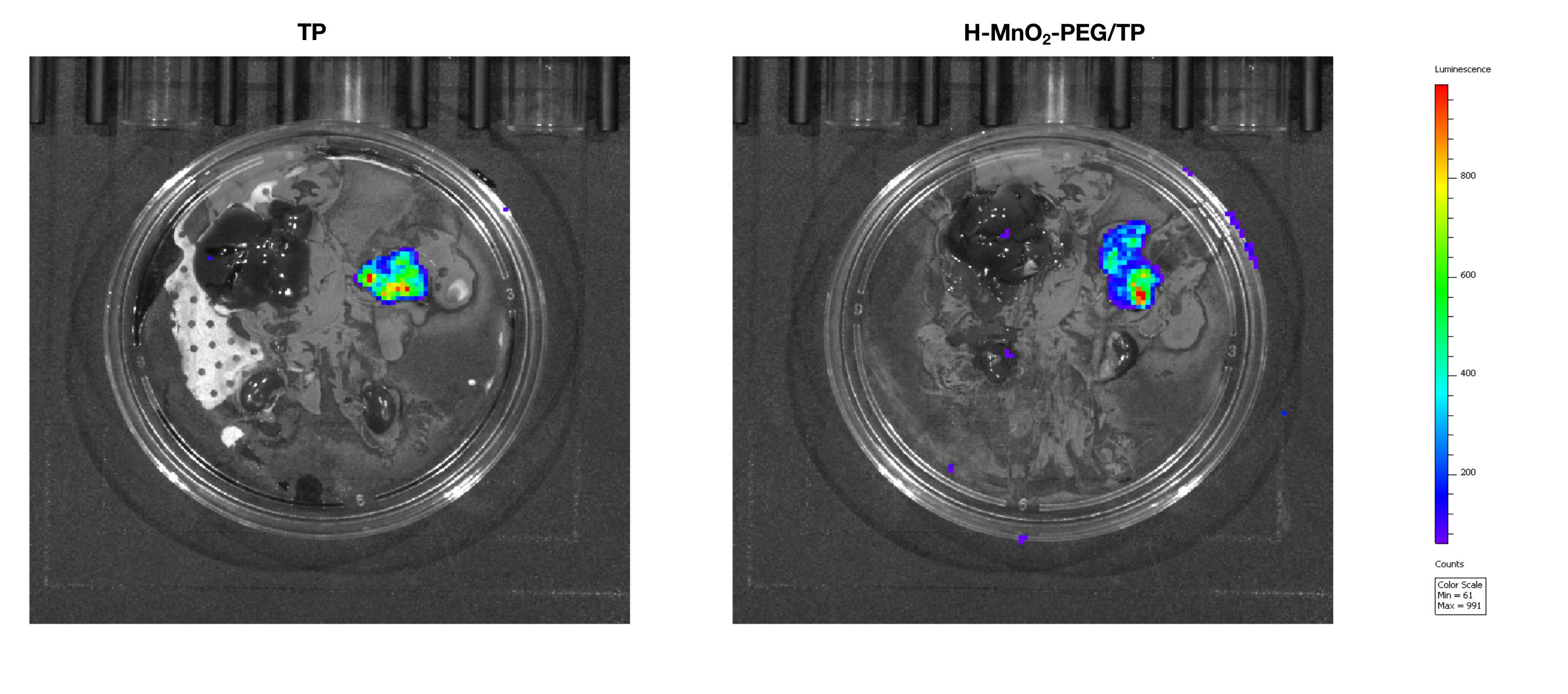

Supplement: Supplementary file 2 — Additional file 2: Figure S2. The in vivo fluorescence imaging of H-MnO2-PEG/TP in pulmonary metastasis Balb/c mice. [file 12951_2021_901_MOESM2_ESM.tif]

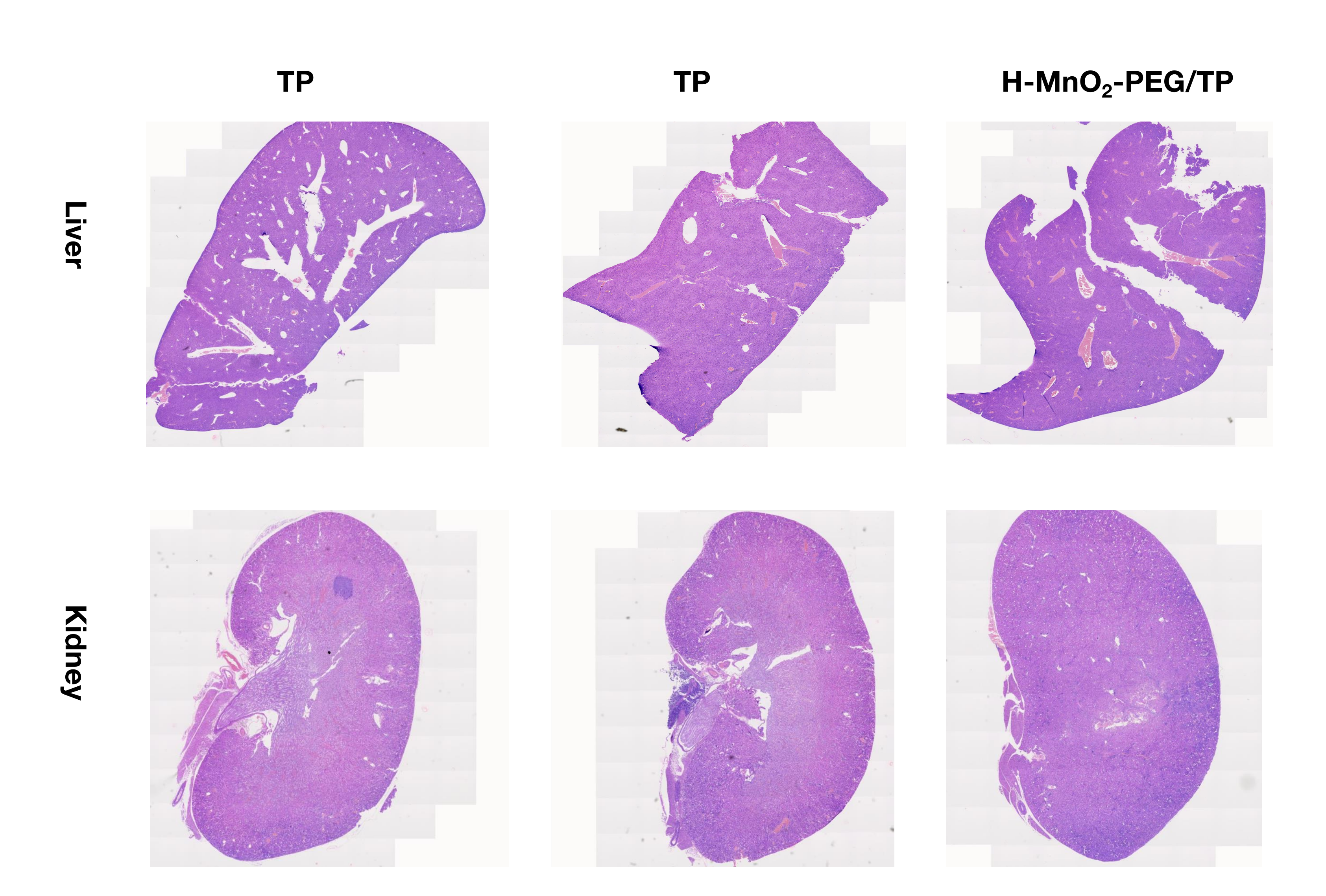

Supplement: Supplementary file 3 — Additional file 3: Figure S3. HE staining of kidney and liver slices. [file 12951_2021_901_MOESM3_ESM.tif]
